# How to Close Wounds of the Larger Arteries

**Published:** 1905-01-28

**Authors:** 


### HOW TO CLOSE WOUNDS OF THE LARGER ARTERIES.

It would be of manifest advantage if some means could be devised for permanently occluding small wounds in the large arteries without destroying the patency of the vessels and so causing a risk of gangrene in the parts which they supply with blood. Suture of the vessel wall has been suggested and even successfully practised in a few instances; but experience has shown that it is not always possible to rely upon the sutures retaining their hold against the repeated strain of the pulse, and for this reason suture is hardly available except for wounds of the smaller arteries, and, possibly, for mere punctures in the larger vessels, such, for instance, as may be caused by the inadvertent use of a needle. A method is suggested by Dr. Brewer in last month's issue of *Annals of Surgery* of occluding the wound by wrapping a strip of adhesive elastic plaster round the wounded artery. The details of the procedure which he has successfully carried out on dogs are as follows:—The wounded artery is isolated for some distance both above and below the wounded point; pressure being made on the vessel above the wound so as to arrest the flow of blood, the artery is cleansed of all blood by the use of a small pledget of gauze moistened with ether; as the ether evaporates the vessel wall is left clean and dry. A small strip of the specially prepared plaster is then passed round the vessel and compressed until it adheres firmly, and forms a tubular sheath to the wounded vessel. The pressure is then removed so as to allow blood to flow through the artery. The vessel is watched for a few minutes to see that the plaster remains firmly adherent after pulsation has reappeared, and the wound is closed. So far as the experiments on dogs are concerned they appear to have been successful on the whole, though in one instance the wound became septic and the animal died of secondary hæmorrhage on the thirteenth day. The principles, if not the minute technique, of Dr. Brewer's method seem to suggest certain limited possibilities of application in man, and they are therefore worthy of consideration.

